# Long‐lasting effects of chronic exposure to chemical pollution on the hologenome of the Manila clam

**DOI:** 10.1111/eva.13319

**Published:** 2021-11-27

**Authors:** Mariangela Iannello, Marica Mezzelani, Giulia Dalla Rovere, Morgan Smits, Tomaso Patarnello, Claudio Ciofi, Lisa Carraro, Luciano Boffo, Serena Ferraresso, Massimiliano Babbucci, Sandro Mazzariol, Cinzia Centelleghe, Barbara Cardazzo, Claudio Carrer, Maurizio Varagnolo, Alessandro Nardi, Lucia Pittura, Maura Benedetti, Daniele Fattorini, Francesco Regoli, Fabrizio Ghiselli, Stefania Gorbi, Luca Bargelloni, Massimo Milan

**Affiliations:** ^1^ Department of Biological, Geological, and Environmental Sciences University of Bologna Bologna Italy; ^2^ Department of Life and Environmental Sciences Polytechnic University of Marche Ancona Italy; ^3^ Department of Comparative Biomedicine and Food Science University of Padova Legnaro Italy; ^4^ Department of Biology University of Florence Sesto Fiorentino Italy; ^5^ Associazione “Vongola Verace di Chioggia” Chioggia Italy; ^6^ c/o Magistrato alle Acque di Venezia Ufficio Tecnico Antinquinamento Laboratorio CSMO Padova Italy; ^7^ Societa’ Agricola Kappa S. S. di Varagnolo Maurizio E. C. Chioggia Italy

**Keywords:** ecotoxicology, hologenome, host‐microbiota interactions, phenotypic plasticity, *Ruditapes philippinarum*

## Abstract

Chronic exposure to pollutants affects natural populations, creating specific molecular and biochemical signatures. In the present study, we tested the hypothesis that chronic exposure to pollutants might have substantial effects on the Manila clam hologenome long after removal from contaminated sites. To reach this goal, a highly integrative approach was implemented, combining transcriptome, genetic and microbiota analyses with the evaluation of biochemical and histological profiles of the edible Manila clam *Ruditapes philippinarum*, as it was transplanted for 6 months from the polluted area of Porto Marghera (PM) to the clean area of Chioggia (Venice lagoon, Italy). One month post‐transplantation, PM clams showed several modifications to its resident microbiota, including an overrepresentation of the opportunistic pathogen *Arcobacter* spp. This may be related to the upregulation of several immune genes in the PM clams, potentially representing a host response to the increased abundance of deleterious bacteria. Six months after transplantation, PM clams demonstrated a lower ability to respond to environmental/physiological stressors related to the summer season, and the hepatopancreas‐associated microbiota still showed different compositions among PM and CH clams. This study confirms that different stressors have predictable effects in clams at different biological levels and demonstrates that chronic exposure to pollutants leads to long‐lasting effects on the animal hologenome. In addition, no genetic differentiation between samples from the two areas was detected, confirming that PM and CH clams belong to a single population. Overall, the obtained responses were largely reversible and potentially related to phenotypic plasticity rather than genetic adaptation. The results here presented will be functional for the assessment of the environmental risk imposed by chemicals on an economically important bivalve species.

## 1 INTRODUCTION

Highly polluted marine areas provide the opportunity to study the response of local populations to environmental variables. In fact, chronic exposure to pollutants may exert strong constant selective pressure on natural populations of aquatic animals. To cope with such pressure, marine organisms may respond by significantly altering the transcriptome profile of different organs, especially those involved in detoxification (*e*.*g*. liver). In the case of acclimatization, when phenotypic plasticity is responsible for the organism response to environmental stressors, transcriptional changes can be entirely reversible, temporary and repeatable (Hodgins‐Davis & Townsend, 2009; Lopez‐Maury et al., 2008; Whitehead & Crawford, 2006; Whitehead et al., 2017). In the case of adaptation, selection on genetic polymorphisms with permanent alterations of the transcriptome may be observed in exposed animals (Bélanger‐Deschênes et al., 2013; Bozinovic & Oleksiak, 2010; Nacci et al., 2016; Reitzel et al., 2014; Whitehead et al., 2010, 2012). A third level of response is observed when epigenetic modifications (*i*.*e*. DNA methylation and histone modifications), without alteration of the DNA sequence, occur (Aluru et al., 2011; Rey et al., 2016; Suarez‐Ulloa et al., 2015). Alterations of the epigenome might be heritable, which makes epigenetic response as intermediate between acclimatization and genetic adaptation. In addition to these three levels of genomic response to environmental variables (phenotypic plasticity, genetic adaptation and epigenetic modifications), a fourth genomic component, the microbiome, is becoming increasingly recognized. In the last two decades, the relationships between resident microbial organisms and their eukaryotic hosts have been extensively explored, demonstrating that the host microbiota plays a vital role in host development and health, as well as in its responses to environmental perturbations and anthropogenic‐induced changes in the marine realm (Gootenberg & Turnbaugh, 2010; McFall‐Ngai et al., 2013; Bourne et al., 2016; Apprill, 2017; Milan et al., 2018; Milan, Maroso, et al., 2019). Concerning bivalve species, recent studies demonstrated that bivalve microbiota is shaped by the environment and strongly suggested the existence of long‐term association between bivalve hosts and specific bacterial taxa (Dupont et al., 2020; Milan, Maroso, et al., 2019; Milan, Smits, et al., 2019; Offret et al., 2020).

The relative role of these four dimensions of response to environmental factors largely remains an open question. Here, we present the case of Manila clam (*Ruditapes philippinarum*) inhabiting two different sites within the Venice lagoon. The Manila clam is a filter‐feeding organism living in seafloor sediment that represents the most important species for commercial clam landings in Europe and one of the major aquacultured species in the world. Its remarkable ability to adapt to a wide range of environmental conditions makes it an excellent model species for the study of adaptation to environmental changes, as well as for biomonitoring impacted marine areas (e.g. Ji et al., 2015; Matozzo et al., 2010; Moschino et al., 2010; Sacchi et al., 2013; Wang, Li, et al., 2010; Wang, Zhou, et al., 2010). Native to the Asian coasts of the Indian and Pacific oceans, the Manila clam was introduced to Europe a few decades ago (Chiesa et al., 2017; Cordero et al., 2017; Flassch & Leborgne, 1992; Utting & Spencer, 1992).

Localized areas characterized by high levels of pollution have been observed within the Venice lagoon since the early 1970s, when the rapid industrialization process of the Porto Marghera (PM) area led to severe chemical contamination of soil, groundwater and sediments. Despite the progressive reduction of industrial activities, the PM area still shows high levels of pollutants, specifically dioxins, halogenated compounds and trace metals, as testified by the ban on shellfish harvesting enforced by local authorities (DGR3366/2004; MODUS 4; Milan et al., 2015, 2018). In the past few years, we studied how such a contrasting situation in the same water body affects natural populations of the Manila clam. In a previous study, we showed specific transcriptome and biochemical profiles in clams collected in PM that reflected molecular signatures of chemical stress (Milan et al., 2011, 2013, 2015). Subsequently, we explored the role of clam microbiota in response to environmental variables, demonstrating the recurrent presence of putatively detoxifying bacterial taxa in PM clams and the potential for host‐microbial synergistic detoxifying actions (Milan et al., 2018). Finally, in a short‐term common garden experiment, we observed that clams from PM showed a significantly less pronounced response to experimental copper exposure than clams originating from the clean area of Chioggia (CH), despite having measured similar concentrations of this metal in both PM and CH (Matozzo et al., 2013; Milan et al., 2016). The latter study suggested that chronic exposure to pollution (i.e. dioxin and PCBs) may influence how animals respond to other environmental stressors. Building on all the evidence discussed thus far, we set up a field experiment to test the hypothesis that chronic exposure to chemical pollutants may have substantial effects on the Manila clam hologenome (microbiome and host transcriptome) long after removal from the contaminated site. To further implement a highly integrative biological approach and increase the ecological relevance of the investigated responses, we performed transcriptome and microbiome analyses, supplemented by an evaluation of biochemical and histological profiles. Overall, this study provides new evidence of long‐lasting effects of chronic exposure to pollution in a species of particular commercial interest. The results obtained will be functional to the scientific community and regulatory agencies to assess and predict the environmental risks imposed by chemicals on natural and farmed populations.

## 2 MATERIALS AND METHODS

### 2.1 Sampling activities

**FIGURE 1:**
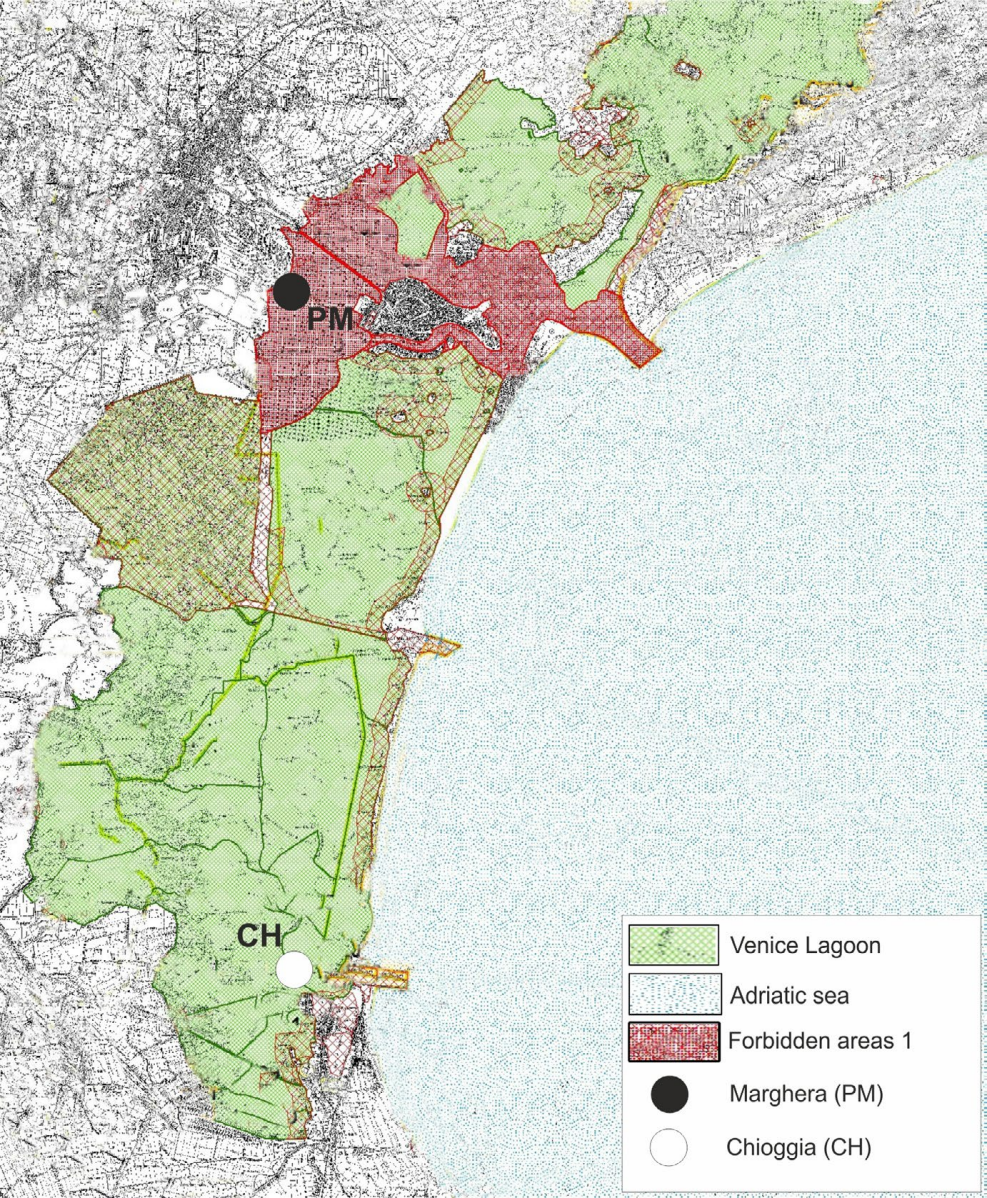
Map of the Venice lagoon indicating the Manila clam sampling sites: Marghera (PM) and Chioggia (CH). Some individuals collected in PM were transplanted to CH and collected at 1 and 6 months post‐transplantation

Approximately 200 adult Manila clams were collected in late January 2016 (T0) in a polluted site in inner Porto Marghera (PM_T0) and in the farming area of Chioggia (CH_T0), which is characterized by the absence of detectable chemical contamination (Figure 1). An additional 500 Manila clams collected in PM at T0 were transplanted to the CH site and placed into cages (100 × 100 × 25 cm) made of plastic netting. To provide duplicate assays, two cages were utilized per sampling time. These cages were partially buried into and anchored to the sediments. Investigated CH clams were sampled in one of the most important Chioggia clam farming sites and placed in cages in the same manner as the transplanted PM clams.

A total of 200 PM transplanted clams and 200 native CH clams were collected after one month (early March‐T1: PM_T1 and CH_T1) and after 6 months (early August‐T2: PM_T2 and CH_T2) post‐transplantation (see details in Table S1). To increase homogeneity across samples, Manila clams of similar size (3.5–3.8 cm shell length) and weight (9–12 g) were included in the study. After each sampling (T0, T1 and T2), clams were transferred to the laboratory, and for each sample set, the digestive glands of five individuals grown in different cages (2 and 3 clams/each) were harvested for transcriptome and microbiome analyses. In addition, for each sampling time/site, haemolymph and digestive glands were rapidly removed from 30 specimens, pooled in 10 samples (each consisting of tissues from three specimens), frozen in liquid nitrogen and maintained at −80°C for biochemical analyses. An aliquot of haemolymph was also immediately processed for lysosomal membrane stability, and another aliquot was fixed with Carnoy’s solution (3:1 methanol, acetic acid) for the microscopic evaluation of micronuclei frequency. In addition, 10 replicates, each composed of whole tissues of at least 15 organisms, were also harvested and stored at −20°C for chemical analyses. The geographical coordinates, number of individuals and experimental analyses performed for each sampling site are summarized in Table S1 and Table 1.

**TABLE 1:** Summary of the experimental plan and analyses performed on Manila clam samples. Genetic analyses comparing CH and PM populations were performed considering RNA‐Seq data of 15 PM and 15 CH individuals collected at different sampling times (T0, T1, T2)

Sampling time	Sampling area	Sample ID	Sampling date	Analysis
TO	Chioggia (CH)	CH_T0	January 2016	RNA‐Seq (for transcriptomic and genetic analyses) and microbiota analyses (5 individuals/population) biomarkers (10 pools/population) histological analyses (5 individuals/population) chemical analyses (10 pools/population)
	Marghera(PM)	PM_T0		
Transplantation of 500 Manila clams collected in PM at T0 to the CH site				
T1	Chioggia (CH)	CH_T1	March 2016	RNA‐Seq (for transcriptomic and genetic analyses) and microbiota analyses (5 individuals/population) biomarkers (10 pools/population) histological analyses (5 individuals/population) chemical analyses (10 pools/population)
		PM_T1		
T2	Chioggia (CH)	CH_T2	August 2016	RNA‐Seq (for transcriptomic and genetic analyses) and microbiota analyses (5 individuals/population) biomarkers (10 pools/population) histological analyses (5 individuals/population) chemical analyses (10 pools/population)
		PM_T2		

### 2.2 Environmental parameters and chemical analyses

Water temperature and salinity were measured on‐site at each sampling time with a field thermometer and a hand‐held refractometer. Additional information on environmental parameters (salinity, temperature, dissolved oxygen, chlorophyll) was collected through monitoring stations located in close proximity to the two sampling areas from the Water Regional Authority (publicly available; Table S1), allowing for definition of the annual trends of different environmental variables in the two areas of the investigated samples. Aliphatic hydrocarbons (C10–C40), polycyclic aromatic hydrocarbons (PAHs), halogenated persistent organic pollutants and trace metals (As, Cd, Cr, Cu, Hg, Ni, Pb, V, Zn) were analysed in the whole body mass of clams by conventional procedures, based on gas chromatography with a flame ionization detector or mass spectrometry, high‐performance liquid chromatography with diode array (DAD) and fluorimetric detection and atomic absorption spectrophotometry techniques, as described and validated elsewhere (Regoli et al., 2019). Details on the analytical methods and procedures for quality assurance/quality control are given in File [Link], [Link]. Additional information about bioaccumulation and concentrations of chemicals in the sediment has been provided by the Regional Monitoring Plan MODUS 4.

### 2.3 Gene expression analyses

Total RNA was extracted from the digestive glands of five individuals for each investigated group (CH_T0, PM_T0, CH_T1, PM_T1, CH_T2 and PM_T2) using the RNeasy Mini Kit (Qiagen) according to the manufacturer’s instructions. Each extraction cycle included a sterility control. The same extracted RNA was examined for both gene expression (RNA‐Seq) and microbiota analyses (16S). cDNA libraries were constructed from five individuals for each sampling site using a SureSelect Strand‐Specific mRNA Library (Agilent Technologies) according to the manufacturer's protocol (details are reported in File [Link], [Link]). Library pools were sequenced using a HiSeq 4000 sequencer (Illumina) with a 50‐bp single‐end approach, yielding a total of 765,204,350 reads (details are reported in Table S1, sequences available in NCBI SRA; BioProject PRJNA612420).

A *de novo* version of the *R*. *philippinarum* digestive gland transcriptome was assembled starting from a library of two individuals (collected in PM and CH) sequenced a HiSeq 2500 sequencer (Illumina) with a 100‐bp paired‐end approach (details are reported in File S1). A total of 53,476 transcript contigs were obtained (reported in File S2) and used as the reference transcriptome for RNA‐Seq read mapping. Transcriptome annotation was performed by a Blastx similarity search on SwissProt (UniProt), *Homo sapiens* protein Ensembl database, *Danio rerio* protein Ensembl database and *Crassostrea gigas* protein Ensembl database (*E*value < 0.0001). Of 53,476 unique sequences, 32,711 (61%) showed at least one significant match. Details and the annotation of each contig are reported in Table S1. Adapter trimming and read mapping were carried out on CLC Genomics Workbench v.10.1.1 (CLCbio). Detailed information is reported in File S1 and Table S1. Raw gene counts obtained from mapping (see File S1) were used as input in EdgeR (Robinson et al., 2010) to analyse differential gene expression. Samples were grouped according to sampling area/time and transcripts showing less than two counts per million (CPM) in 4 out of 5 samples per group (in all investigated groups) were discarded. Samples were then normalized by EdgeR using the Trimmed Mean of M‐values (TMM) method. After filtering, a total of 29,303 transcripts were kept for subsequent analysis. A likelihood ratio test was carried out in EdgeR to assess differentially expressed genes (DEGs) between PM and CH at T0, T1 and T2, with a significant fold change (FC) threshold set to >1.5 and a false discovery rate (FDR) set to <0.05. To detect differences in the transcriptome profiles of PM and CH across the three time points, we used Next maSigPro (Nueda et al., 2014), a software program developed to analyse time series experiments (see details in File S1).

Gene set enrichment analysis (GSEA) was employed to identify enriched functional categories. To focus on biological processes (BP) and KEGG pathways that were consistently differentially regulated in PM and/or consistently correlated to chemical concentrations in our previous studies (Milan et al., 2013, 2015, 2016), a panel of 27 gene sets was evaluated, which included pathways and BP related to cell cycle, DNA repair, detoxification processes, protein turnover, response to external stimulus, energy metabolism, immune response and cell processes (full list of gene sets is reported in Table 2). An additional gene set named the ‘Porto Marghera Gene Set’ consisted of the six transcripts that were differentially expressed in PM clams compared to every other sampling site and across different seasons in our previous study (*Glutathione sulfotransferase theta*‐*1*, *Sulfotransferase 1C4*, *AChE*, *Cytochrome P450 3A9*, *Steryl sulfatase* and *Peroxisomal 2*,*4*‐*dienoyl*‐*CoA reductase*; Milan et al., 2013, 2015) (see details in File S1).

**TABLE 2:**
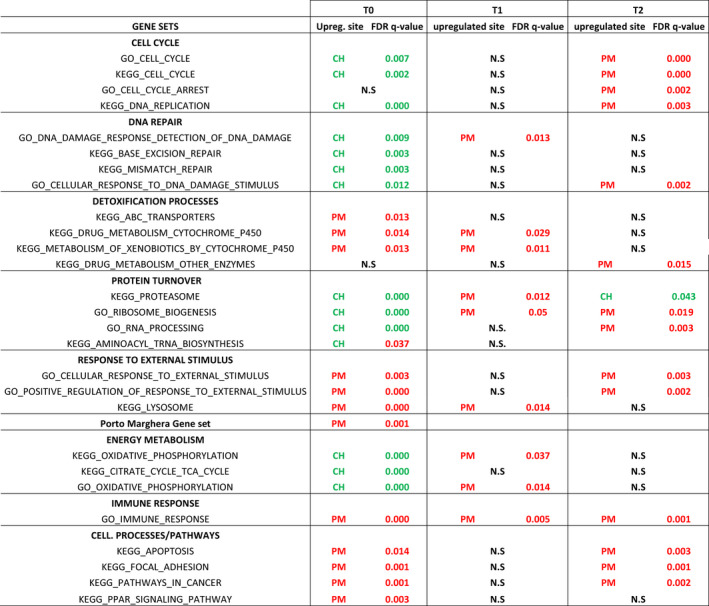
GSEA performed at each sampling time. A panel of 28 gene set were analysed. Columns T0, T1 and T2 report the upregulated site and corresponding FDR‐*q* value for the significant gene sets identified for at least one sampling time. N.S.: not significant. Red and green colour indicates up‐ and down‐regulated molecular pathways, respectively

### 2.4 Biomarkers analyses

Biomarkers in clam tissues were measured using standardized protocols (Bocchetti et al., 2008) that included spectrophotometric determination of acetylcholinesterase (AChE) in haemolymph and metallothioneins (MTs), acyl‐CoA oxidase (ACOX), antioxidant defences (catalase; glutathione S‐transferases, GST; Se‐dependent and sum of Se‐dependent and Se‐independent glutathione peroxidases, GPx‐H2O2 and GPx‐CHP; glutathione reductase, GR), malondialdehyde (MDA) in digestive glands; gas chromatographic assay of total antioxidant capacity (TOSC) towards peroxyl radicals (ROO·) and hydroxyl radicals (HO·) in digestive glands; lysosomal membrane stability and genotoxic damage (micronuclei (MN) frequency) in clam haemocytes. Detailed protocols are given in File S1. Statistical analyses of data were performed using IBM SPSS Statistics software. Homogeneity of variance was checked by Cochran C, and analysis of variance (ANOVA) was applied to chemical data and biomarker responses to evaluate the effect of site time and their interaction, followed by Tukey HSD *post hoc* tests. The data on micronuclei frequency were analysed by the nonparametric Kruskal–Wallis test.

### 2.5 Histological analyses

Tissue samples collected from 5 Manila clams for each site/sampling time were placed in 10% neutral‐buffered formalin for at least 48 h, routinely processed and embedded in paraffin. Specimens were cut into 5‐μm‐thick sections and stained with haematoxylin and eosin for standard light microscopic evaluation. To investigate tissues morphology and the possible presence of organ lesions, a complete histological examination was performed on dorso‐ventral section of the middle of the body of clams (Howard et al., 2004). Each section included the visceral mass, gonad, gills and mantle.

### 2.6 Microbiota characterization

The same extracted RNA for gene expression (RNA‐Seq) was used for microbiota characterization. A single sterility control pool was created by mixing 1 μl of each sterility control, and the resulting pool was included in the microbiome analysis. The RNA integrity number (RIN) index was calculated for each sample using Agilent 2100 Expert software. To reduce experimental biases in RNA‐Seq analysis due to poor RNA quality, only RNA samples with an RIN >7 were further processed.

For microbiota analyses, 1 μg of RNA was reverse transcribed to cDNA using the Superscript IV Kit (Invitrogen, Life Technologies). cDNA was diluted to 0.2 ng/μl and amplified in a 50 μl reaction that included 5 μl of diluted DNA and 1.5 μl of both the reverse and forward primers (10 μM) that specifically target the V3‐V4 gene region of the bacterial 16S rRNA, as described by Milan et al. (2018). Libraries were then pooled together based on their concentrations, and the final pool was quantified using a Bioanalyzer 2100 (Agilent Technologies) and sequenced by BMR Genomics with an Illumina MiSeq (2x300). Microbiome sequencing generated approximately 6 million reads (passing filters), averaging approximately 100,000 reads per pool (sequences available in the NCBI Sequence Read Archive (SRA) https://www.ncbi.nlm.nih.gov/sra; SUB7159366). Raw reads were first analysed using CLC Genomic WorkBench version 10.1.1 to select high‐quality sequences. Reads with a Phred score lower than 20 were discarded (https://www.qiagenbioinformatics.com/). Sequences were uploaded in QIIME 2 (Quantitative insights into microbial ecology; Bolyen et al., 2019) using DADA2 (Callahan et al., 2016) to filter adaptors, low‐quality sequences and to merge forward and reverse reads of each fragment and obtain high‐quality representative sequences. After the quality‐filter step, read merging and removal of chimaeric fragments, a total of 1,528,388 reads were retained, represented by 1,802 features. Sequence alignment was performed using MAFFT software (Katoh & Standley, 2013) and then classified using the Python library Scikit‐Learn on QIIME2. Taxa assignment was carried out using the SILVA‐trained database (132 update release). To normalize our analysis, all samples were rarefied to 20,688 reads. The statistical analysis was performed using CALYPSO software (Zakrzewski et al., 2017), using the features table produced in QIIME2. For each time point, all samples were organized by principal coordinate analysis (PCoA), and a two‐way ANOVA was carried out to identify different taxa between experimental groups at OTUs level (*p*‐value < 0.05).

### 2.7 Genetic data analysis

Genetic analysis were performed considering 15 PM and 15 CH individuals, regardless of sampling time. Quality of the RNA‐Seq reads was assessed using the FastQC quality control tool v0.11.5, before being mapped an annotated version of the *R*. *philippinarum* genome assembly (F. Ghiselli unpublished data) using Rsubread v2.4.2 (Liao et al., 2019). Parameters for mapping reads were as follows: maxMismatches = 5, nTrim5 = 6, unique = FALSE, nBestLocations = 3. The resulting BAM files were used for variant calling with Freebayes v1.2.0 (Garrison & Marth, 2012), a tool for Bayesian haplotype‐based genetic polymorphism discovery. Parameters used for the variant calling were as follows: use‐best‐n‐alleles = 4, min‐alternate‐count = 3, min‐alternate‐fraction = 0.05, min‐mapping‐quality = 20. The resulting VCF (Variant Call Format) file was further filtered to retain variants present in at least 80% of samples using Bcftools v1.11, according to the following criteria for biallelic SNPs and insertions/deletions (indels): min‐alleles = 2, max‐alleles = 2, type = snp/indel, min‐af = 0.01, exclude‐min‐quality<20, exclude‐max‐missing>0.2.

Next, genotypes for SNPs and indels (in 0/1 format) were extracted from the filtered VCF file using the Genome Analysis ToolKit (GATK) v4.1.9.0, and genotype counts by population (*n* = 2; 15 individuals for each population) were used as input for the BayPass package v2.2, a population genomics software primarily aimed at identifying genetic markers subjected to selection and/or associated to population‐specific covariates (Gautier, 2015). Candidate loci under selection that were identified by BayPass as being significantly contrasted (*p*‐value < 0.001 and Bayes Factor > 20) between CH and PM were then functionally annotated using Annovar (Wang, Li, et al., 2010). For all SNP loci, pairwise (CH vs PM) FST statistics were estimated using the *adegenet* (v 2.1.3) and *hierfstat* (v 0.5‐7) R packages.

## 3 RESULTS

### 3.1 Environmental parameters and chemical analysis

Negligible differences in temperature, salinity and chlorophyll concentrations were recorded between PM and CH (Table S1) at each sampling time (T0, T1 and T2). A significant increase in water temperature between T0‐T1 and T2 was observed, which was expected because the first two samplings were carried out in the winter, while the last sampling was carried out in the summer. Concentrations of trace metals, PAHs and aliphatic hydrocarbons (C10‐C40) measured in whole soft tissues of clams are reported in File S3. At the beginning of the translocations, higher tissue levels of trace metals Cd (1.033 ± 0.15 µg/g dry weight, d.w), Fe 1451 ± 358.2 µg/g d.w.) and Pb (2.079 ± 0.35 µg/g d.w.) were measured in organisms from PM compared with those of CH (0.41 ± 0.08, 715.61 ± 88.56, 0.95 ± 0.03 µg/g d.w., respectively; File S3). Eventually, a significant decrease in Cd was measured for clams from PM during the translocation period, finally reaching a concentration similar to that which was measured for specimens from CH at the end of the experiment (T2) (File S3, two‐way ANOVA). A similar pattern of variation was also observed for Hg and Pb: even after 6 months of translocation, the levels of Pb in organisms from PM were still higher than those measured in clams from CH (File S3). Tissue levels of aliphatic hydrocarbons (C10‐C40, ranging between 161.1 ± 18.01 and 208.66 ± 49.04 µg/g d.w.) and PAHs (205.50 ± 21.51–468.30 ± 142.20 µg/g d.w.) were comparable between organisms from different sites at all sampling times.

### 3.2 Transcriptome analysis

Gene expression profiles were analysed either separately for each time point or as a time series. The complete DEG list is reported in File S6. A pairwise comparison of PM and CH clams at T0 showed over 100 DEGs (FDR‐adjusted *p*‐value < 0.05; FC > 1.5); with an upregulation in PM clams for several genes involved in xenobiotic metabolism (*CYP3A4*, *CYP27B1*, *SULT1C4*, *SULT2A1* and *AQP8*; see gene abbreviation list), immune response and inflammation (*PLCL*, *C1QL4*, *GVIN1*, *HMCN1*, *FBCD1*, *CLEC*, *C1QT3*, *MRC1*, *SERPINB1* and *COLEC12*), protein turnover and lysosome (*CATL1*, *DENND3*, *MIB2*, *MYLIP*, *VAMP7*, *PRSS16*, *ARSB* and *ARSI*). After one month in the same environment, fewer genes were differentially expressed (25), although some showed dramatic differences (FC > 38 for *SULT1B1* and FC > 400 for *HSP7012A*). After 6 months, the number of significant DEGs increased to 60, represented mainly by genes encoding proteins involved in cellular stress response mechanisms, such as apoptosis (*BIRC7* and *IAP1*), immune response (*CLEC*, *IFI44L*, *HAAF*, *HMCN1* and *C1QL4*), lysosome function and protein turnover (*CATL1*, *ARSB* and *ANKIB1*). PM samples also showed a significant upregulation of the putative *heavy metal*‐*binding protein HIP* (FC > 90). No genes were found commonly differentially expressed at T0, T1 and T2, while 2 DEGs coding for putative *Cathepsin L (CATL1)* and *Oncoprotein*‐*induced transcript 3 protein* (*OIT3*) were commonly found upregulated in PM clams at the first and the last sampling time.

To consider the contributions of all genes, a GSEA was carried out. The results obtained are summarized in Table 2. Gene sets involved in the cell cycle, RNA processing, ribosome biogenesis and energy metabolism were significantly enriched in CH clams compared to PM clams at T0, while the pattern was reversed after translocation (significant upregulation in PM samples at T1 and/or T2). Gene sets involved in xenobiotic metabolism followed an opposite trend, resulting in upregulation in PM at T0 and one month after translocation. In addition to precompiled gene sets, we also tested a custom set composed of six transcripts that were consistently upregulated in PM according to our previous research, irrespective of either the sampling season or sampling year (Milan et al., 2013, 2015). This analysis showed significant upregulation in PM only at T0. Gene sets involved in cellular stress (i.e. apoptosis, cancer pathways, cellular response to external stimuli, positive regulation of response to external stimuli) were upregulated in PM clams at T0 and T2 but not at T1, confirming the trend observed for DEGs, while immune response was upregulated in PM at all sampling times.

**FIGURE 2:**
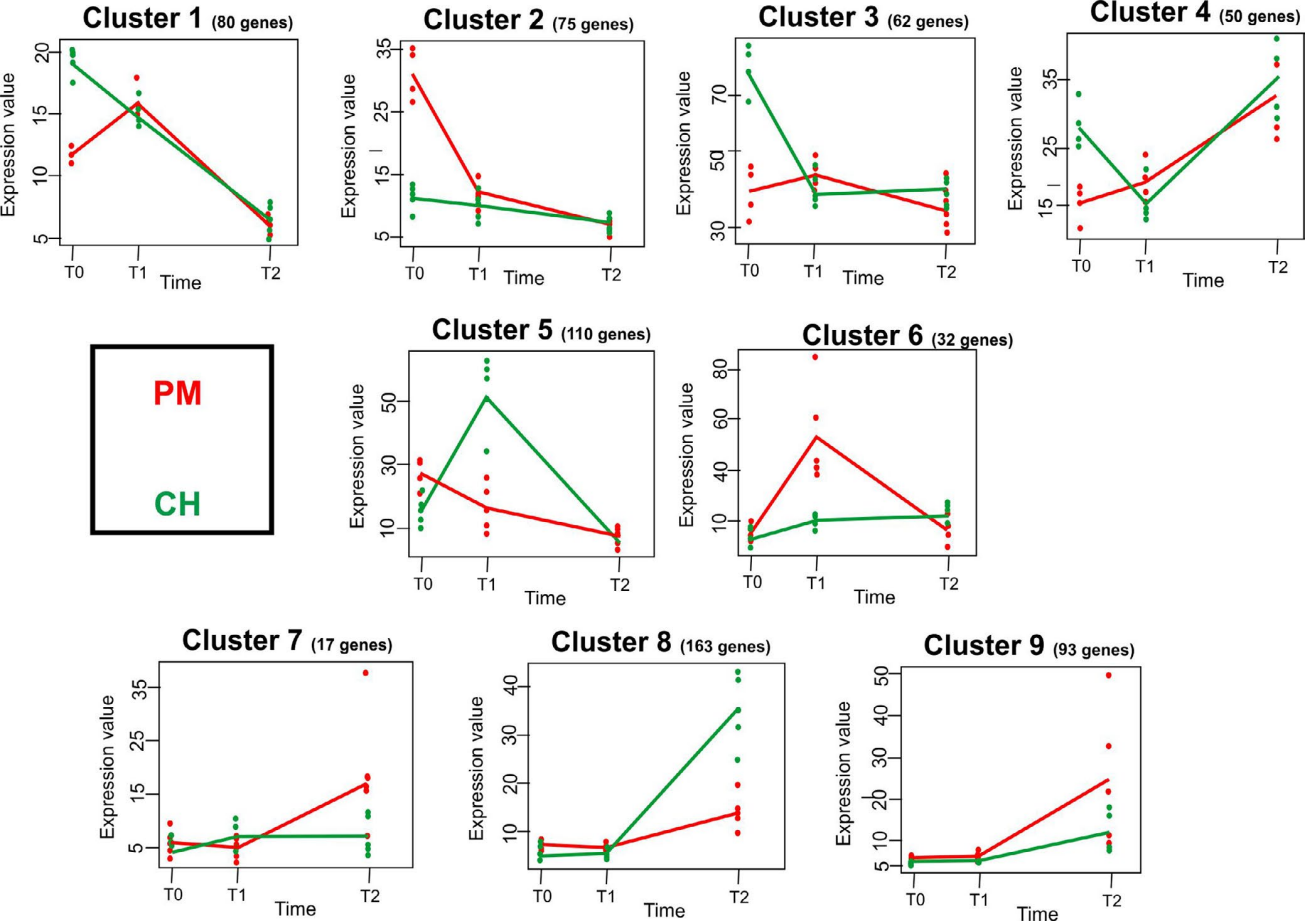
Clusters identified by time series analyses. The number of significant genes belonging to each cluster is also reported

Transcriptome profiles were also evaluated in a time‐course analysis considering the whole transcriptome, which identified nine gene clusters (Figure 2; the complete lists of significant genes and functional annotations for each cluster are reported in File S6), summarized by three different temporal patterns. The first cluster (gene clusters 1–4) included genes that were differentially transcribed at T0 but that showed similar profiles in PM and CH clams at T1 and T2. Genes in clusters 1–4 largely overlapped with those already described as differentially expressed at T0 (*e*.*g*. cluster 2 consisted of genes involved in xenobiotic metabolism, whereas clusters 1 and 4 included transcripts involved in metabolic pathways and ribosome biogenesis). A second pattern was represented by gene clusters 5 and 6, which mostly included genes involved in the immune response, and showing the most relevant differences at T1. Finally, a third pattern included clusters 7, 8 and 9, where profiles of PM and CH clams were different at T2. Most genes comprised of clusters 7–9 play key roles in cellular response to stimuli, apoptosis regulation, stress response, cell cycle and immune response.

### 3.3 Biochemical analyses

Variations in biomarker responses according to site and period are reported in Table 3 and File S4. Micronuclei (MN) frequency was significantly affected by site, period and the interaction of the two: the high values measured at T0 in native PM clams decreased after one month of translocation to the low frequency detected in CH clams. Lysosomal membrane stability revealed significant variations considering the sampling period; conversely, AChE activity was affected by site, with consistently lower values in PM at T1. Significant variations in metallothionein (MT) levels and acyl‐CoA oxidase (ACOX) activity were measured, considering both site and period (Table 3): after one month of translocation, higher values of MT were observed in bivalves from PM compared to CH, and such levels decreased and were similar in both sites at the end of the translocation experiment. In contrast, ACOX activity was inhibited in native clams from PM before translocation compared to those from CH, and such differences levelled out after translocation. Several biomarkers of oxidative stress (catalase, glutathione S‐transferase, malondialdehyde and total oxyradical scavenging capacity •OH) highlighted significant variations within periods.

**TABLE 3:** Results of two‐way analysis of variance for the biological responses in *R. philippinarum*

VARIABLE	SITE			TIME			SITE × TIME		
	dF	*F*	*p*	dF	*F*	*p*	dF	*F*	*p*
Mn	1	24.23	<0.001	2	30.19	<0.001	2	26.12	<0.05
NRRT	1	0.53	N.S.	2	19.26	<0.001	2	6.24	<0.05
AchE	1	5.81	<0.05	2	1.39	N.S.	2	0.081	N.S.
MT	1	5.34	<0.05	2	21.59	<0.001	2	1.95	N.S.
ACOX	1	21.29	<0.01	2	4.79	<0.05	2	4.8	<0.05
CAT	1	2.65	N.S.	2	10.9	<0.001	2	2.82	N.S.
GST	1	0.00	N.S.	2	10.44	<0.01	2	0.293	N.S.
Gpx Cu	1	1.31	N.S.	2	2.27	N.S.	2	0.83	N.S.
Gpx H2O2	1	0.2	N.S.	2	1.86	N.S.	2	2.38	N.S.
GR	1	0.19	N.S.	2	1.49	N.S.	2	1.01	N.S.
MDA	1	0.08	N.S.	2	4.3	<0.05	2	0.23	N.S.
TOSC ROO•	1	0.19	N.S.	2	2.42	N.S.	2	0.738	N.S.
TOSC •OH	1	0.39	N.S.	2	11.98	<0.05	2	1.82	N.S.

Abbreviations: AchE, acetylcholinesterase; ACOX, acyl‐CoA oxidase; CAT, catalase; df, degree of freedom; F, F test; Gpx Cu, sum of Se‐dependent and Se‐independent glutathione peroxidases; Gpx H_2_O_2_, Se‐dependent glutathione peroxidases; GR, glutathione reductase; GST, glutathione‐S transferases; MDA_,_ levels of malondialdehyde; Mn, micronuclei frequency; MT, levels of metallothioneins; N.S., not significant; NRRT, neutral red retention time; P, probability level; TOSC HO•, total oxyradical scavenging capacity toward hydroxyl radical; TOSC ROO•, total oxyradical scavenging capacity toward peroxyl radical.

### 3.4 Histology

A total of 5 Manila clam samples for each site were histologically examined. The cross section taken included the whole body of each individual. Normal microscopic structures were found, and no anatomical abnormalities or histological lesions were underlined.

### 3.5 Microbiota characterization

**FIGURE 3:**
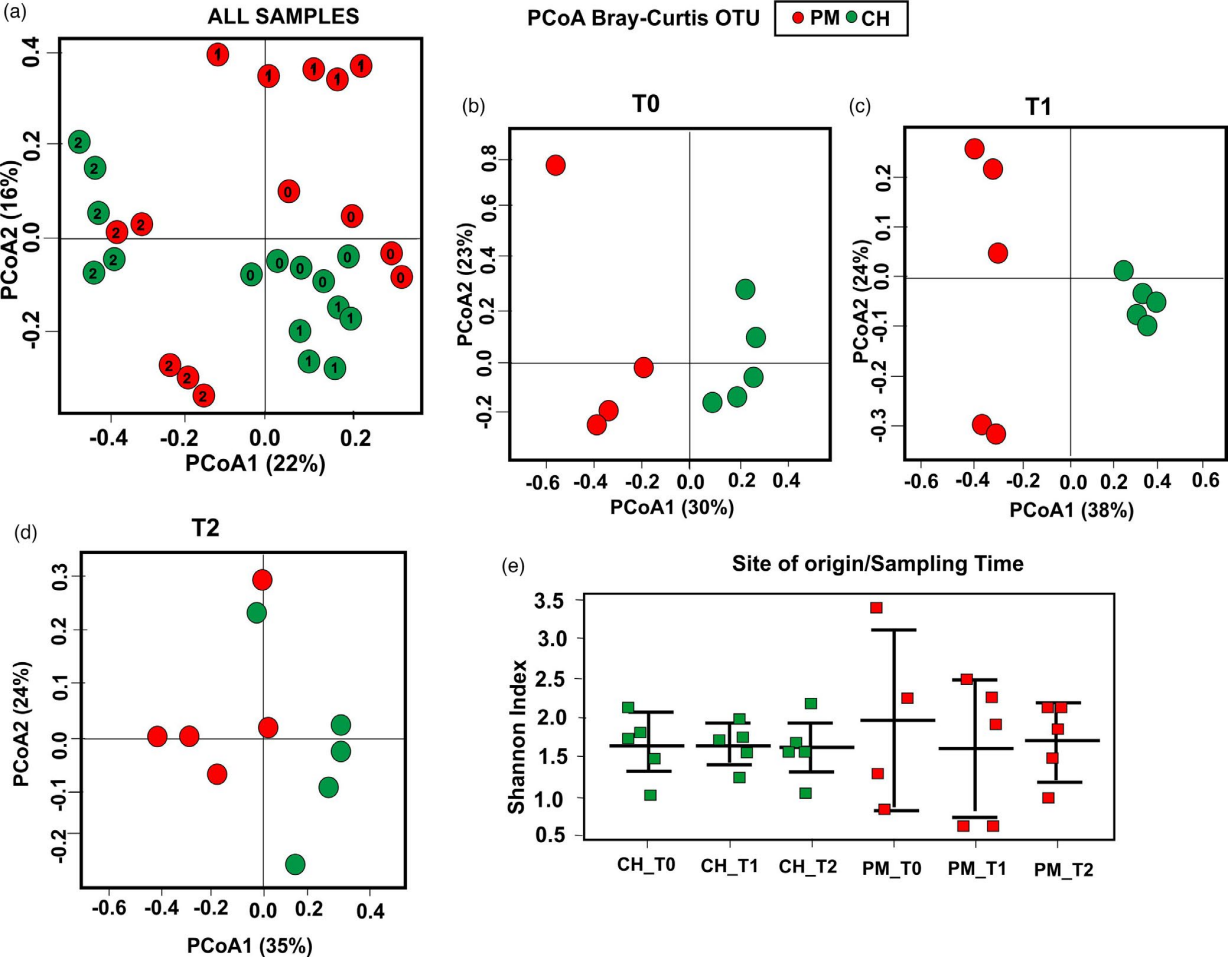
Principal coordinate analysis (PCoA) and Shannon's index of diversity. Different colours indicate the different origin sites (PM = red; CH = green). (a) PCoA obtained considering all investigated samples. Sampling times are indicated by different numbers (0 = T0; 1 = T1; and 2 = T2); (b, c and d) PCoA performed separately for each sampling time. e) Shannon's index of diversity reported for each sampling time and site of origin

Clam‐associated bacterial communities consisted primarily of Proteobacteria (59%), Tenericutes (26.1%), Chlamydiae (5.4%), Spirochaetes (4.8%) and Bacteroidetes (3.4%). The most abundant classes were Alphaproteobacteria (47.6%), Mollicutes (26.1%) and Gammaproteobacteria (6.1%), while *Mycoplasma* (phylum Tenericutes) was the most represented genus (25.9%): the overall distribution of phyla and classes for each pool is reported in File S7. A principal coordinates analysis (PCoA; Bray–Curtis distance) that considered all samples showed a separation along the first axis (PC1 = 22% explained variation) between samples collected in winter (T0 and T1) from those collected in summer (T2). However, while microbial profiles of CH clams collected in winter (T0 and T1) appeared very similar, the microbial composition of PM clams one month after transplantation showed significant changes from native clams (Figure 3a). A PCoA performed separately at each sampling time showed a clear separation along the first axis between PM and CH clams at both T0 and T1 (Figure 3b,c). Six months after translocation, separation was less clear, though still evident (Figure 3d). Shannon’s index of diversity highlighted extreme across‐individual variation in PM clams at T0 compared to CH native clams. Such variation decreased one month after transplantation in Chioggia, reaching a similar degree of variance at the end of the experiment, as observed in CH clams at all sampling times (Figure 3e). To further explore changes in microbiota between PM and CH clams before and after transplantation, a one‐way ANOVA was performed for each sampling time (the complete list of significant taxa at each sampling time is reported in File S5). At T0, 16 and 6 overrepresented OTUs were observed in PM and CH, respectively. However, the highest number of differentially represented taxa was found at T1, with a total of 46 overrepresented OTUs in PM clams and 19 OTUs in CH clams. Six months after transplantation, the number of differentially abundant OTUs was smaller (17 OTUs overrepresented in PM clams and 4 OTUs in CH clams), as already observed in the PCoA.

### 3.6 SNP calling and genotyping

RNA‐Seq data of 15 CH individuals and 15 PM individuals consisted of 366.4 M and 361.7 M reads, of which 63.5% and 57.6% were mapped to the clam genome assembly, respectively. FeatureCounts was able to assign 67.5% of CH samples and 65.9% of PM samples to an annotated element of the genome. Of the 1,066,809 total variants called (1,022,447 were SNPs), 360,055 biallelic SNPs and 9,721 indels were retained after filtering and further analysed. Genetic differentiation expressed as FST (estimated according to Weir and Cockerham (1984)) was −0.00037 (bootstrap confidence interval with *p*‐value 0.99). Contrast analysis using BayPass showed that no indels were significantly contrasted between the two sites, while 17 SNPs were highly significantly contrasted (File S8). Annotation of the variants using ANNOVAR indicated that 10 SNPs were located in annotated regions, whereas the remaining 7 lacked annotation information (File S5). None of the 17 contrasting SNPs were located in the DEGs, which had been identified in the transcriptomic analysis.

## 4 DISCUSSION

### 4.1 Chemical contamination and its effects on the clam hologenome

Levels of chemical pollutants were confirmed to be higher in clams living in PM than in CH, especially for trace metals. Detected concentrations were within the range of previously reported values for this area and typical of areas exposed to anthropogenic disturbance (Boscolo et al., 2007; Moschino et al., 2012). Concentrations of PCDD/Fs, PCB and HCB were not measured in this study, as such data were available from the Regional Monitoring Program (MODUS 4) for the same year and the same locations (PM and CH), further highlighting higher levels of these organic pollutants in PM clams (Table S1). Likewise, PCDD/Fs, PCB and HCB were not analysed at T1 and T2 because the content of these compounds in clam tissue rapidly decreases to 1/10 of the original level after just one week in uncontaminated water (Milan et al., 2016; Raccanelli et al., 2008). The different chemical burden in PM and CH clams reflected significant variations in transcriptional, biochemical and cellular responses and microbial community composition. At the functional level, haemocyte parameters were the most sensitive in revealing differences between native clams from PM and CH: MN frequency, as a marker of genotoxic damage, clearly highlighted the negative effects of pollutants present in PM, along with the impairment of lysosomal membrane stability. This evidence suggests potential effects on cell physiology, intracellular turnover, immune responses, degradation and eliminations of pathogens, in which bivalve haemocytes are primarily involved (Mezzelani et al., 2021). Moreover, the inhibition of ACOX activity in native PM clams, which catalyses the first reaction of β‐mitochondrial oxidation of fatty acids, may be related to changes in the metabolism of energy resources driven by several classes of environmental pollutants (Mezzelani et al., 2021). At the transcriptome level, most DEGs at T0 overlapped with those observed in previous studies (Milan et al., 2013, 2015, 2016). In analogy with biochemical and cellular biomarker results, functional analysis of DEGs, genes in clusters 1–4 and GSEA all showed a clear indication of upregulated genes in PM involved in xenobiotic metabolism, protein turnover, lysosome, immune response and tissue damage in PM clams. In addition to SULT, GST and CYP450 playing key role in detoxification and widely discussed in previous studies (Milan et al., 2013, 2015, 2016), the upregulation of *AQP*‐*8* (FC > 4) should be also highlighted as it was already found permanently overexpressed in PM under controlled conditions and differentially regulated compared to CH clams in response to heavy metal exposure study (Milan et al., 2016). This protein belongs to a family of integral membrane proteins functioning as water‐selective channels and playing an important role in cellular osmotic balanced volume regulation (Agre et al., 2002; King et al., 2004). Recent studies demonstrated an increased expression of AQP‐encoding genes under copper and lead exposures, suggesting they might have a role in preventing the deleterious effects of heavy metals on the cellular ionic and volume regulation (e.g. Boyle et al., 2013; Rocha & Souza, 2012). Conversely, organisms living in the clean environment (CH) were characterized by a general upregulation of normal metabolic pathways, including oxidative phosphorylation and citrate cycle suggesting higher energy budget in CH clams compared to PM. Downregulation of these key biological process in PM clams could be related to reduced energy input from food because of reduced feeding activity, with the net effect of energy trade‐offs that may affect population dynamics by altering growth performance, development, reproductive output and immune function (Akberali & Trueman, 1985; Sendra et al., 2021; Sussarellu et al., 2016; Widdows, 1985). In the future, experiments measuring ecophysiological parameters (e.g. growth, respiration) under controlled conditions might provide confirmation to this hypothesis.

Overall, clam‐associated bacterial communities were similar to those found in a previous study (Milan et al., 2018), indicating a rather species‐specific composition of microbial communities in the clam hepatopancreas. The large variability in bacterial diversity observed across different PM individuals may indicate less stable, potentially dysbiotic bacterial communities associated with clams challenged by high chemical contamination, although such a hypothesis would require a larger number of individual microbiome data to be tested. Overall, the evidence supports the hypothesis that Manila clams chronically exposed to chemical pollution in PM respond consistently across different years at all investigated biological levels (transcriptome, biochemical and microbiome).

### 4.2 Same season, no chemical pollution: The clam hologenome one month post‐transplantation

In accordance with the rapid decrease of PCDD/Fs, PCBs and HCB to 1/10 of the original level after just one week in uncontaminated water (Raccanelli et al., 2008) and decreases of trace metal concentrations one month after transplantation, the observed pattern of multiple responses to chemical pollution almost disappeared, suggesting that the specific response to chemical stress highlighted is reversible and temporary. The only possible exceptions were the overexpression of SULT1B1 and the inhibition of AChE activity at the catalytic level. In addition, several lines of evidence (DEGs, GSEA, gene clusters 5–6) suggested enrichment in immune‐related functions for upregulated genes in PM clams. Even more significant is the difference in the hepatopancreas microbiota between the two groups of animals, despite one month of cohabitation in the same environment. While variations in microbial diversity were less marked across individuals (Figure 3e), PM clams had a substantial number of bacterial taxa with different abundances, particularly for *Arcobacter* spp. (10 overrepresented taxa), a potentially opportunistic pathogen that is frequently associated with unhealthy animals (Fan et al., 2013; Lokmer & Wegner, 2015; Tanaka et al., 2004) and has already been described in our previous study (Milan et al., 2018). Several of the other overrepresented taxa in PM clams have been frequently associated with polluted environments, including Porto Marghera. These include *Pseudomonas* (8 OTUs overrepresented in PM at T1), *Acinetobacter* (3 OTUs overrepresented in PM) and *Zoogloea* (Gao et al., 2016; Milan et al., 2018; Mollaeia et al., 2010; Ouyang et al., 2016; Sohn et al., 2004; Wasi et al., 2013). Key functions of microbiota in the physiology and health of vertebrate species have been widely investigated (Ruby et al., 2004), as well as how genetics, diet and other external factors influence the gut microbial community. Knowledge of bivalve microbiota is still underdeveloped, with the exception of a few studies related to either public health or mortalities that involve bivalve species of commercial interest (Romalde et al., 2013; Li et al., 2018; King et al., 2019; Li et al., 2019; Milan, Maroso, et al., 2019). It has been recently reported that the microbiota of filter‐feeding animals may be significantly modified through the acquisition of microbial species directly from the environment, as well as by seasonal environmental fluctuations in chemical–physical parameters that characterize marine and lagoon environments (Beleneva & Zhukova, 2009; Dubilier et al., 2008; Meisterhans et al., 2016; Milan et al., 2018; Tanaka et al., 2004). However, stable host–microbiota associations have also been reported (e.g. Baldi et al., 2013; Desriac et al., 2014; Freese & Schink, 2011; Harris, 1993; Moriarty, 1997; Wegner et al., 2013; Zurel et al., 2011). In this respect, it has been shown that the microbiota composition of the clam hepatopancreas is site‐ and season‐specific (Milan et al., 2018), but consistent within sites and seasons even across different years (Milan, Maroso, et al., 2019; Milan, Smits, et al., 2019). One month in the same environment was not sufficient to equalize the PM clam microbiota to that of CH animals, although it likely altered the microbial community, leading to the emergence of opportunistic pathogens such as *Arcobacter* spp. Such modifications to the resident microbiota may be related to the upregulation of several immune genes in the PM clams (T1), which could represent the host response to the increased abundance of deleterious bacteria. In particular, overexpression of *BDEF*, *RUNX1* and *FKBP1A* (cluster 5) suggests specific responses to pathogens. *BDEF* is a well‐known AMP with remarkable microbicidal activity against bacteria in bivalve species (Zhao et al., 2010); RUNX represents a transcription factor involved in the immune response (Song et al., 2019; Zhang et al., 2014), while *FKBP1A* belongs to a family of proteins implicated in the response to infectious pathogen (Chen et al., 2011). Other DEGs that suggest a targeted response against pathogenic microorganisms are those encoding mannose receptors (*MRC1* and *MRC2*), *lactose*‐*binding lectin l*‐*2* (*CLEC10A*) and *perlucin*‐*like protein* (*PLCL*).

### 4.3 Different seasons and different stressors: The clam hologenome 6 months after transplantation

After 6 months of translocation, only Pb tissue levels were still significantly higher in PM clams. These clams also exhibited a significant upregulation of the putative *heavy metal*‐*binding protein (HIP)* (FC > 90), which has a potential role in metal binding and detoxification, as a carrier of divalent cations in the plasma (Hattan et al., 2001; Yin et al., 2005). In addition to chemicals, seasonality exerts a profound effect on the Manila clam hepatopancreas transcriptome, its associated microbiome, along with a marked modulation of several biological parameters normally used as biomarkers (Milan et al., 2013, 2018). Several results from the present study confirm this evidence. The summer season is expected to induce higher oxidative challenge in clams due to higher water temperatures, higher metabolic demand and lower oxygen availability. An additional challenge is represented by energy investment in reproduction, as the Venice lagoon clams reproduce between June and September. Evidence from oxidative stress markers appears to support this scenario. The effect of season is quite evident on the microbial community composition irrespective of the clam origin, as the first axis in the PCoA clearly separated summer samples from winter samples (Figure 3a). However, significant differences still existed between PM and CH after 6 months, which was potentially related to the strong resilience of the clam microbiota, but also to a different response to seasonality in the two groups of animals. As the host is able to modulate resident microbiota, persistent differences in its physiology may translate into differences in microbial communities. The transcriptome appeared to be markedly different during the summer in the CH and PM clams, and the time‐course analysis clearly showed three clusters (7–9) where a large set of genes were not differentially expressed at T0 and T1 but displayed a divergent pattern at T2. Among these genes, variations in 17 of them were statistically significant (FDR *p* < 0.05), and nearly all (16) were upregulated in CH individuals, which mostly encoded either stress‐related proteins or pro‐survival anti‐apoptotic factors, such as three members of the IAP repeat‐containing protein family (*BIRC7*, *BIRC7A* and *IAP1*). BIRCs have also been overexpressed at the RNA level in bivalve species under sulphide and hypoxia stress (Wang et al., 2019). The others are represented by chaperonin proteins (Files S5 and S6) belonging to different HSP families (*HSP70*, *HSP40* and *HSP20*) or HSP‐interacting proteins. HSPs are integral to the cell response to different types of stress (*e*.*g*. thermal and oxidative), as demonstrated in the bivalve *Crassostrea gigas* (Liu et al., 2019). All this evidence may suggest that PM clams have a lower ability to respond to environmental/physiological stressors (higher temperatures and reproduction) even after a relatively long acclimation to a pollutant‐free area. Similar evidence has been observed for the response to copper exposure, which was less marked in PM clams after removal from a contaminated site (Milan et al., 2016), although the time frame (three weeks) was shorter than that reported here. In coming years, it will be fundamental to perform further analyses aimed at confirming (or rejecting) the hypothesis that the observed temporal variation in CH and PM clam hologenome is mostly attributable to season effects.

### 4.4 Genetic analysis

Analysis of anonymous genetic variants from 2bRAD sequencing data obtained in a previous study from 62 CH individuals and 56 PM individuals collected in 2009 and 2011 (Milan, Maroso, et al., 2019; Milan, Smits, et al., 2019) demonstrated high connectivity between the two investigated sites. Neither global Fst nor outlier analysis identified significant genetic divergence between time replicates of population samples for the two sites. PM and CH are separated by a small geographic distance and are located respectively in the Central and Southern sub‐basin, which are contiguous. Substantial water exchange in both directions (southward and northward) between the two sub‐basins is ensured by strong tidal currents, north (*bora*) and southeast winds (*scirocco*) (see figure 5 in Solidoro et al., 2004). Venice lagoon hydrodynamics, coupled with the relatively long reproductive season (June–September) and the duration of the planktonic larval phase (8–10 days), likely provides the conditions for high dispersal ability of Manila clams.

While all these biotic and abiotic factors prevent genetic divergence between CH and PM clams, it might be possible that selection linked to the peculiar conditions in PM acts on few, specific genetic loci. Analysis of a much larger set of SNPs (>360,000), which might also be functionally relevant, showed a limited number of significantly divergent loci, which could be under selection. However, caution should be exerted as SNP calling was carried out on RNA‐Seq data and on a limited number of samples (15) per site. The proteins encoded by the significant loci are apparently not related with response to xenobiotics. In fact, their putative function is either related to cell metabolism (ctg972_210115, *glucose dehydrogenase*) or other basal cell mechanisms (e.g. ctg1086_147409, *A disintegrin and metalloproteinase with thrombospondin motifs 6*; ctg271_183388 *Cyclin*‐*dependent kinase 11B*). The most significant locus (ctg2639_92644) encodes a protein with a pumilio domain. Pumilio/Puf family RNA binding proteins are highly conserved across the animal kingdom and are involved in the post‐transcription regulation of gene expression in a broad array of BP (Nishanth & Simon, 2020).

## 5 CONCLUSIONS

In conclusion, this study confirmed that different external factors (chemical pollution and environmental variation) induce an organismal response that is repeatable as it is consistent with biochemical, transcriptome and microbiome evidence reported in previous studies. The clam response to chemical pollution appears largely reversible, suggesting that it may be largely attributable to phenotypic plasticity. Limited evidence was found for genetic adaptation, with a small number of loci potentially under divergent selection. Such evidence remains to be further explored in a larger set of samples, possibly with temporal replicates. A common garden experiment on F1 clams originating from controlled crosses (CHxCH, PMxPM, CHxPM) should provide the ultimate proof for the role of genetics in the clam response to chemical pollution. While the specific response to xenobiotics is transient, chronic exposure to pollutants in PM clams had long‐lasting effects on the animal hologenome. The hepatopancreas‐associated microbiota still showed a different composition after 1 and 6 months, and, more importantly, the responsiveness to other environmental factors appeared to be different (be less pronounced) in PM clams. In contrast, only small variations between biomarker responses of PM and CH clams were observed after 6 months; this finding confirms the complexity of functional responses in the Manila clam, further highlighting the importance of applying a multidisciplinary approach that enables us to provide an exhaustive view of the overall biological status of natural populations. It remains to be investigated whether such long‐term effects may be explained by epigenetic modifications or other mechanisms, for example, pollutant‐mediated impoverishment of energy reserves. It would also be interesting to further investigate the interactions between the host and associated microbiota, focusing on changes in the microbial community and immune response in the host or the effects of chemicals on host microbiota. Such interactions may have short‐term consequences on the host but also long‐term effects, as observed in this study.

### 5.1 Gene abbreviation list

*CYP3A4*: *Cytochrome P450 3A24*; *CYP27B1*: *Cytochrome P450 27C1*; *SULT1C4*: *Sulfotransferase 1C4*; *SULT2A1*: *Bile salt sulfotransferase*; *AQP8*: *Aquaporin*‐*8*; *PLCL*: *Perlucin*‐*like protein*; *C1QL4*: *Complement C1q*‐*like protein 4*; *GVIN1*: *Interferon*‐*induced very large GTPase 1*; *HMCN1*: *Hemicentin*‐*1*; *FBCD1*: *Fibrinogen C domain*‐*containing protein 1*; *CLEC*: *C*‐*type lectin domain family*; *C1QT3*: *Complement C1q tumour necrosis factor*‐*related protein 3*; *MRC1*: *Macrophage mannose receptor 1*; *SERPINB1*: *serpin B1*; *COLEC12*: *Collectin*‐*12*; *CATL1*: *Cathepsin L1*; *DENND3*: *DENN domain*‐*containing protein 3*; *MIB2*: *E3 ubiquitin*‐*protein ligase MIB2*; *MYLIP*: *E3 ubiquitin*‐*protein ligase MYLIP*; *VAMP7*: *Vesicle*‐*associated membrane protein 7*; *PRSS16*: *Putative serine protease*; *ARSB*: *Arylsulfatase B*; *ARSI*: *Arylsulfatase I*; *SULT1B1*: *Sulfotransferase family cytosolic 1B member 1*; *HSP7012A*: *Heat shock 70 kDa protein 12A*; *BIRC7*: *Baculoviral IAP repeat*‐*containing protein 7*; *IAP1*: *Death*‐*associated inhibitor of apoptosis 1*; *IFI44L*: *Interferon*‐*induced protein 44*; *HAAF*: *Hemagglutinin*/*amebocyte aggregation factor*; *ANKIB1*: *ankyrin repeat and IBR domain containing 1*; *BDEF*: *Big defensin*; *RUNX1*: *Runt*‐*related transcription factor 1*. *FKBP1A*: *FK*‐*506 binding protein*; *CLEC10A*: *lactose*‐*binding lectin l*‐*2*; and *PLCL*: *perlucin*‐*like protein (PLCL* *)*.

## AUTHOR CONTRIBUTIONS

Massimo Milan, Luca Bargelloni, Stefania Gorbi, Claudio Ciofi, Francesco Regoli and Tomaso Patarnello conceived and designed the project. Marica Mezzelani, Alessandro Nardi, Lucia Pittura, Giulia Dalla Rovere, Maurizio Varagnolo, Luciano Boffo, Massimo Milan and Stefania Gorbi performed transplantation and *R*. *philippinarum* sampling activities. Sandro Mazzariol and Cinzia Centelleghe performed histological analyses. Stefania Gorbi, Marica Mezzelani, Alessandro Nardi, Lucia Pittura, Maura Benedetti and Daniele Fattorini performed chemical (heavy metals and PAHs) and biochemical analyses. Claudio Carrer performed PCDD/F and PCB chemical analyses. Lisa Carraro, Giulia Dalla Rovere, Massimiliano Babbucci and Barbara Cardazzo carried out 16S statistical analyses (16S). Mariangela Iannello, Massimo Milan, Fabrizio Ghiselli and Serena Feraresso performed gene expression analyses. Morgan Smits, Serena Ferraresso and Luca Bargelloni carried out SNP detection and genetic analyses. Milan Massimo, Luca Bargelloni, Stefania Gorbi, Francesco Regoli, Marica Mezzelani, Mariangela Iannello and Giulia Dalla Rovere wrote the manuscript and prepared the figures. All listed authors edited the final version of the manuscript. All authors read and approved the manuscript.

## CONFLICT OF INTEREST

The authors declare no conflict of interest.

## Supporting information

File S1

File S2

File S3

File S4

File S5

File S6

File S7

File S8

Table S1

## Data Availability

16S sequence data are deposited in the SRA database (SUB7159366). RNA‐Seq data sequencing files are available in the NCBI Sequence Read Archive (SRA; https://www.ncbi.nlm.nih.gov/sra; BioProject PRJNA612420).
