# Supplementary material for: Long‐lasting effects of chronic exposure to chemical pollution on the hologenome of the Manila clam
Source: Evol Appl. 2021 Nov 27;14(12):2864–80. doi: 10.1111/eva.13319 (PMC8674894; doi:10.1111/eva.13319)
Supplement: Supplementary file 1 — File S1 [file EVA-14-2864-s007.doc]

**Supplementary File S1**

**Methods for chemical analyses, biomarker measurement, gene expression analyses, SNP calling, genotyping and Population genetics data analysis**

***Chemical analyses***

Chemical analyses in clam tissues have been determined according to previous methods with slight modifications (Regoli et al., 2019).

Aliphatic hydrocarbons and halogenated persistent organic pollutants were extracted treating tissues (about 3 g, wet weight) with hexane:acetone (2:1) in a microwave (110°C for 25 min, 800 Watt) (Mars CEM, CEM Corporation, Matthews NC). After centrifugation at 3.000 × *g* for 10 min, the supernatants were purified with solid-phase extraction (Phenomenex Strata-X, 500 mg × 6 mL plus Phenomenex Strata-FL, 1000 mg × 6 mL) and then concentrated using a SpeedVac (RC1009; Jouan, Nantes, France) to dryness. Samples were finally recovered with 1mL of pure GC grade *n*-hexane. Aliphatic Hydrocarbons were determined by gas chromatograph (Perkin Elmer) equipped with an Elite-5 capillary column (30 m × 0.32 mm ID × 0.25 μm-df) and a flame ionization detector (FID). For quantitative determination, the system was calibrated with an unsaturated pair *n*-alkane standard mixture according to EN ISO 9377-3 (Fluka 68281).

Halogenated persistent organic pollutants were analyzed with a GC-MS system (Varian Saturn 2000 ion trap, Agilent Technologies, Santa Clara, CA, USA) using a GC capillary column Zebron (Zebron ZB-5MS, 30m, 0.25mmID, 0.25µ, Phenomenex), applying three different spit-splitless and oven temperature ramp methods for different class of pollutants: 1): BTEX (benzene, toluene, ethylbenzene and xylene congeners); 2): chlorophenols (2,4-dichlorophenol, 2,4,6-trichlorophenol, pentachlorophenol), OCPs (2,4-dichlorophenol, 2,4,6-trichlorophenol, pentachlorophenol, α-lindane, β-lindane, δ-lindane, γ-lindane, α-chlordane, γ-chlordane, 4,4'-DDD, 4,4'-DDE, 4,4'-DDT, aldrin, dichlorobenzidine, dieldrin, endrin, endrin aldehyde, endrin ketone, hexachlorobenzene, methoxychlor, endosulfan I, endosulfan II, endosulfan sulfate, heptachlor, heptachlor epoxide), chlorobiphenyls and PCBs congeners (2-chlorobiphenyl, 3-chlorobiphenyl, 4-chlorobiphenyl, PCB4, PCB8, PCB11, PCB16, PCB17, PCB18, PCB19, PCB28, PCB38, PCB44, PCB46, PCB52, PCB66, PCB77, PCB81, PCB101, PCB105, PCB118, PCB126, PCB128, PCB138, PCB153, PCB156, PCB169, PCB170, PCB172, PCB180, PCB182, PCB187, PCB192, PCB195, PCB203, PCB206 and PCB209); 3): BFRs, including hexabromocyclododecane (HBCD), tetrabromobisphenol A (TBBPA) and polybromodiphenylethers (PBDE28, PBDE47, PBDE100, PBDE99, PBDE154, PBDE153, PBDE183).

The various compounds were determined by the retention time of analytical pure standard solution mix (Supelco 47505-U, BTEX/MTBE; Supelco 43240-U, DM 471 Phenol mix; NIST 1493, PCBs congeners; Supelco 4-8862, Aroclor mix 2; Fluka 36989, PCBs congeners; Supelco 46845-U, Pesticide 8081; Supelco 4-0008, Hexachlorobenzene; Supelco 4-0026, 3,3’-dichlorobenzidine; AccuStandard M-1614-CSM, PBDEs congeners of primary interest; Fluka 11223, TBBPA; Aldrich 144762, HBCD), comparing the mass spectra with those of the pure standard compounds and also verifying these by means of the NIST Database (NIST/EPA/NIH Mass Spectra Library version 2.0, National Institute for Standard and Technologies, NIST, Gaithersburg, MD, USA).

For analysis of polycyclic aromatic hydrocarbons (PAHs), about 3 g (wet weight) of tissues were extracted in 10 mL 0.5 M potassium hydroxide in methanol with microwave at 55°C for 20 min (800 Watt) (CEM, Mars System). After centrifugation at 3.000 × *g* for 10 min, the methanolic solutions were concentrated using a SpeedVac and purified with solid-phase extraction (Octadecyl C18, 500 mg × 6 mL, Bakerbond). A final volume of 1 mL was recovered with pure, analytical HPLC gradient grade acetonitrile, and HPLC analyses were carried out in a water and acetonitrile gradient by fluorimetric and diode array detection. The PAHs were identified according to the retention times of an appropriate pure standards solution (EPA 610 Polynuclear Aromatic Hydrocarbons Mix), and classified as low molecular weight (LMW: naphthalene, acenaphthylene, 1-methyl naphthalene, 2-methyl naphthalene, acenaphthene, fluorene, phenanthrene, anthracene) or high molecular weight (HMW: fluoranthene, pyrene, benzo(a)antrhacene, chrysene, 7,12-dimethyl benzo(a)anthracene, benzo(b)fluoranthene, benzo(k)fluoranthene, benzo(a)pyrene, dibenzo(a,h)anthracene, benzo(g,h,i)perylene, indeno(1,2,3,c,d)pyrene).

For trace metals, tissues were dried to constant weight at 60°C and digested under pressure with nitric acid and hydrogen peroxide (7:1) with microwave. Arsenic, cadmium, chromium, copper, iron, manganese, nickel, lead, vanadium, zinc were analyzed by atomic absorption spectrophotometry, with flame (Varian, SpectrAA 220FS) and flameless atomization (Varian SpectrAA 240Z); the mercury content was quantified by cold vapor atomic absorption spectrometry (Cetac QuickTrace M-6100 Mercury Analyzer).

For all of the chemical analyses, quality assurance and quality control were monitored by processing blank and reference standard materials (NIST-2977, National Institute of Standards and Technology; ERM CE477, EC–DG JRC Institute for Reference Materials and Measurements). The concentrations obtained from these SRM analyses were always within the 95% confidence intervals of the certified values. The water content in tissues was determined in all of the samples, and the concentrations were expressed as ng·g^-1^ dry weight (dw) for PAHs and PCBs, while as μg·g^-1^ dw for aliphatic hydrocarbons (C_10_-C_40_) and trace metals.

***Biomarkers analyses***

Detailed procedures for all presented biomarkers have been described elsewhere (Bocchetti et al., 2008). Metallothioneins (MTs) were analyzed in clams digestive glands after acidic ethanol/chloroform fractionation of tissues homogenates, and spectrophotometric quantification using reduced glutathione (GSH) as standard (Viarengo et al., 1997). The activity of peroxisomal Acyl-CoA oxidase (ACOX) in clams digestive glands was measured with a coupled assay following the production of H_2_O_2_ by the oxidation of dichlorofluorescein-diacetate in the presence of an exogenous horseradish peroxidase (Bocchetti et al., 2008). Acetylcholinesterase activity (AChE) was spectrophotometrically assayed in clams hemolymph using the Ellman’s reaction (Bocchetti et al., 2008). Antioxidants defenses were measured in clams digestive glands following standardized assay conditions, at a constant temperature of 18 ± 1°C (Bocchetti et al., 2008; Regoli et al., 2005). Catalase was determined by the decrease in absorbance due to H_2_O_2_ consumption; glutathione peroxidases (GPx) activities were assayed in a coupled enzyme system where β-nicotinamide adenine dinucleotide (NADPH) is consumed by glutathione reductase to convert the oxidized glutathione (GSSG) to its reduced form using hydrogen peroxide or cumene hydroperoxide as substrates, respectively, for the selenium-dependent and for the sum of Se-dependent and Se-independent forms; glutathione S-transferases (GST) were determined following the reaction between GSH and 1-chloro-2,4-dinitrobenzene (CDNB) as substrate; glutathione reductase (GR) activity was measured by the oxidation of NADPH during the reduction of GSSG.

The Total Oxyradical Scavenging Capacity (TOSC) was measured in clam digestive glands by the capability of cellular antioxidants to inhibit the oxidation of α-keto-γ-methiolbutyric acid (KMBA) to ethylene gas in the presence of different forms of oxyradicals, like peroxyl radicals (ROO·) and hydroxyl radicals (HO·) which are artificially generated at constant rate (Regoli and Winston, 1998, 1999). Ethylene formation was determined by gas-chromatographic analyses and TOSC values were quantified from the equation: TOSC = 100-(∫SA/ ∫CA x 100), where ∫SA and ∫CA are the integrated areas calculated under the kinetic curve produced during the reaction course for respective sample (SA) and control (CA) reactions. For all the samples, a specific TOSC (normalized to content of protein) was calculated by dividing the experimental TOSC values by the relative protein concentration contained in the assay and determined by the Lowry method with Bovine Serum Albumin (BSA) as standard (Regoli and Winston, 1999).

Lysosomal membrane stability (NRRT) was evaluated after the haemocytes incubation on a glass slide with a freshly prepared Neutral Red (NR) working solution (2µl/ml filtered sea water from a stock solution of 20 mg NR dye dissolved in 1 ml of dimethyl sulfoxide) and microscopically examined at 20 min intervals to determine the time at which 50% of cells had lost into the cytosol the dye previously taken up by lysosomes.

The content of malondialdehyde (MDA) was measured in homogenates of clams digestive glands derivatized with 1-metyl-2-phenylindole and spectrophotometrically determined after calibration against a malondialdehyde standard curve (Bocchetti et al., 2008).

The genotoxic effects were evaluated at chromosomal level by the micronucleus test. Micronuclei (MN) frequency was measured in haemocytes, fixed in Carnoy’s solution (3:1 ethanol, acetic acid), dispersed on glass slides and stained with the fluorescent dye 40,6-diamidino-2-phenylindole (DAPI) at 100 ng ml-1. For each specimen, 2000 cells with preserved cytoplasm were scored for the presence of micronuclei, defined as round structures, smaller than 1/3 of the main nucleus diameter, on the same optical plan and clearly separated from it (Nigro et al., 2006).

***Gene expression analyses***

*Library preparation:* The cDNA libraries were constructed using a Sure Select Strand-Specific mRNA Library (Agilent Technologies). Briefly, before fragmentation, oligo d(T) beads were used to purify poly(A) mRNA from total RNA. First-strand cDNA was synthesized from the fragmented mRNA using random hexamer primers, and the cDNA libraries were prepared in accordance with the Illumina protocol. After a purification step, the libraries were quantified with a Qubit Fluorometer (Invitrogen, Carlsbad, CA, USA) and pooled together according to their relative concentrations. The concentration and quality of the pool was assessed by Agilent 2100 Bioanalyzer.

*Mapping:* Adapter trimming and reads mapping were carried out on CLC Genomics Workbench v.10.1.1 (CLCbio, Aarhus, Denmark). Trimmed RNA-Seq reads of each sample were mapped on the reference *R. philippinarum* transcriptome using CLCbio with the following parameters: mismatch cost 2, insertion cost 3, deletion cost 3, length fraction 0.8, and similarity fraction 0.8. The 68-78% of the total reads were successfully mapped. Due to technical problem one sample collected in PM_T0 were discarded from gene expression and microbiota analyses.

*Transcriptome assembly:* FastQC tool (http://www.bioinformatics.babraham.ac.uk/projects/fastqc) was used to perform a quality check of the raw reads. Low quality reads and Illumina adapters were removed using Trimmomatic version 0.36 (Bolger et al. 2014), with the following parameters: TruSeq3-PE.fa: 1:30:10, leading 30, trailing 30, slidingwindow 15:33, minlen 70. Low abundant kmers (C < 5) were filtered out with the trim-low-abund.py script from khmer version 2.1.1 (Crusoe et al. 2015). In order to obtain a comprehensive transcriptome, filtered reads were assembled combining different tools and k-mer sizes. More in detail, we obtained 10 de novo assemblies with the following methods: Trinity v2.4.0 (Grabherr et al. 2011) with k-mer sizes 21, 25 and 31; Trinity v2.4.0 genome guided—using a draft genome assembly of *R. philippinarum* (unpublished, paper in preparation); Velvet 1.2.10-Oases 0.2.8 (Schulz et al. 2012; Zerbino and Birney 2008) with k-mer sizes 21, 25, 31, 41, 61 and 81. All the de novo assemblies were merged together, then we used the EvidentialGene software (http://arthropods.eugenes.org/EvidentialGene/trassembly.html) to identify main transcripts and to remove redundant transcripts and fragment transcripts. This combined transcriptome was used as reference in the following analyses. Evaluation of the completeness was performed with BUSCO v3 (Simão et al. 2015) against the Metazoa ortholog database, as implemented in gVolante (Nishimura et al. 2017), with default parameters.

*Next maSigPro:* to define the regression model we considered a quadratic regression mode (degree=2). We set counts=true to apply the generalized linear models option with negative binomial distribution, as recommended for next generation sequencing series. Differentially expressed genes were identifies using a False Discovery Rate = 0.05, computed with the Benjamini and Hochberg method (MT.adjust=BH). Stepwise regression was executed using the backward method, with pvalue=0.05 and R-squared of 0.6. Genes whose profiles were different between PM and CH were grouped in clusters using the function k.mclust, that compute an optimal number of clusters based of similar profiles.

*Gene set enrichment analysis:* A ranking value was assigned to each gene based on EdgeR lrt p-value as follows: i) logFC>0, score = 1-pval; ii) logFC<0, score = -(1-pval). Enrichment analysis was carried out by setting Enrichment statistic = Weighted, Normalization mode = meandiv and Number of permutation= 1000. To identify the corresponding *R. philippinarum* transcripts composing the “Porto Marghera Gene Set” in the new reference transcriptome here assembled, blastn search have been performed with default parameters considering the DNA microarray probes c14595_AS, P_c17047, P_c28883, N_c12315, c20179_S, 2_c1189_S, 2_c2341_AS (see Milan et al. 2013).

*SNP calling, genotyping and Population genetics data analysis*

RNAseq reads were mapped to the *R. philippinarum* reference genome (Ghiselli et al. in publication) using STAR (Dobin et al., 2013) in two-pass mode. Sorting, read groups assignment and duplicates marking were performed by means of PICARD toolkit (http://broadinstitute.github.io/picard/). SNP discovery and genotyping across all samples was performed simultaneously using HaplotypeCaller tool under standard parameters according to GATK (v 3.7) Best Practices (McKenna et al., 2010; <https://software.broadinstitute.org/gatk/best-practices/>). A first hard filtering step was applied (options --filterExpression "FS>30.0" --filterName FS --filterExpression "QD < 2.0" --filterName QD –genotypeFilterExpression “DP<5” --genotypeFilterName LowDP --setFilteredToNocall) to filter out variants with QualByDepth (QD) < 2.0 and FisherStrand (FS) > 30 accounting for variant quality and strand bias, in addition, loci with individual read depth below 5 were set to no-call. A second filtering step (options --filterExpression "AF < 0.10" --filterName LowMAF --filterExpression "AF >0.90" --filterName HighMAF) was applied to keep only SNPs with minor allele frequency (MAF) of at least 10%. At this level, only SNPs were considered and small insertions and deletions were excluded, the maximum allowed fraction of samples with no-call genotype was set to 0.25. Resulting SNPs (28,922) were further filtered to retain only those confirmed by an independent genomic SNP dataset produced from a PoolSeq experiment of Manila clam sampled in different European populations (unpublished data) resulting in 25,309 SNPs (87.5%). Filtered SNPs (25,309) VCF (Variant Call Format) file was transformed to appropriate formats by means of the PGDSpider (version 2.1.1.1) software (Lischer & Excoffier, 2012), and the detection of candidate loci under selection was performed by means of BayeScan 2.1 (Foll & Gaggiotti, 2008).

***References***

Bocchetti, R., Lamberti, C.V., Pisanelli, B., Razzetti, E.M., Maggi, C., Catalano, B., Sesta, G., Martuccio, G., Gabellini, M., Regoli, F., 2008. Seasonal variations of exposure biomarkers, oxidative stress responses and cell damage in the clams, Tapes philippinarum, and mussels, Mytilus galloprovincialis, from Adriatic Sea. Mar. Environ. Res. 66, 24-26.

Frenzilli, G., Nigro, M., Scarcelli, V., Gorbi , S., Regoli, F., 2001. DNA integrity and total oxyradical scavenging capacity (TOSC) in the Mediterranean clams, *Mytilus galloprovincialis*: a field study in a highly eutrophicated coastal lagoon. Aquatic Toxicology 53, 19-32.

Nigro, M., Falleni, A., Barga, I.D., Scarcelli, V., Lucchesi, P., Regoli, F., Frenzilli, G., 2006. Cellular biomarkers for monitoring estuarine environments: transplanted versus native clams. Aquatic Toxicology. 77, 339-347.

Regoli, F., d’Errico, G., Nardi, A., Mezzelani, M., Fattorini, D., Benedetti, M., Di Carlo, M., Pellegrini, D., Gorbi, S. 2019. Application of a Weight of Evidence Approach for monitoring complex environmental scenarios: the case-study of off-Shore platforms. Front. Mar. Sci., 6 (377). doi.org/10.3389/fmars.2019.00377

Regoli, F., Nigro, M., Benedetti, M., Gorbi, S., Pretti, C., Gervasi, P.G., Fattorini, D., 2005. Interactions between metabolism of trace metals and xenobiotic agonists of the Ah receptor in the Antarctic fish *Trematomus bernacchii*: environmental perspectives. Environmental Toxicology and Chemistry 24, 1475-1482.

Regoli, F., Winston, G.W., 1998. Application of a new method for measuring the Total Oxyradical Scavenging Capacity in marine invertebrates. Marine Environmental Research 46(1–5), 439–442.

Regoli, F., Winston, G.W., 1999. Quantification of Total Oxidant Scavenging Capacity of antioxidants for peroxynitrite, peroxyl radicals and hydroxyl radicals. Toxicology and Applied Pharmacology 156, 96-105.

Viarengo, A., Ponzano, E., Pondero, F., Fabbri, R., 1997. A simple spectrophotometric method for metallothionein evaluation in marine organisms: an application to Mediterranean and Antarctic molluscs. Marine Environmental Research 44, 69-84.
