# Supplementary material for: Long‐lasting effects of chronic exposure to chemical pollution on the hologenome of the Manila clam
Source: Evol Appl. 2021 Nov 27;14(12):2864–80. doi: 10.1111/eva.13319 (PMC8674894; doi:10.1111/eva.13319)
Supplement: Supplementary file 3 — File S3 [file EVA-14-2864-s006.docx]

**Chemical concentrations**

Concentrations of trace metals **(1A),** Polyciclic Aromatic Hydrocarbons (PAHs) and Aliphatic Hydrocarbons (HYDs) **(1B),** in the whole tissues of *R. philippinarum* at each sampling time (T0, T1, T2) in the different origin sites (PM and CH). Data are expressed as mean values ± standard deviation, n=5. Distribution of the aliphatic hydrocarbons in all the analysed groups **(1C).** Data are expressed as mean (straight line) + standard deviation (dotted lines). Lower-case letters indicate significant differences of PM site among sampling times; upper-case letters indicate significant differences of CH site among sampling times; asterisks indicate significant differences between sites within the same sampling time. Results of two-way analysis of variance (ANOVA) for the concentrations of chemicals measured in *R. philippinarum*. dF, degree of freedom; F, F test; P, probability level; N.S., not significant **(1D).**

**1A**


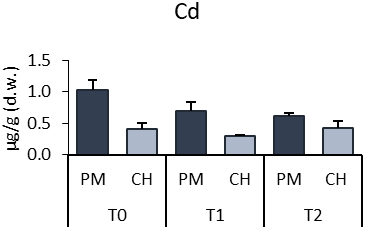


a

b

b

*

*


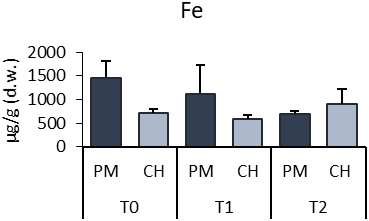


*


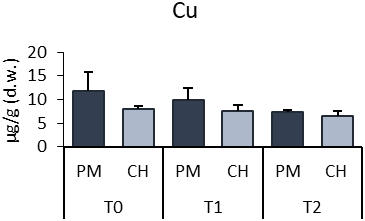


*


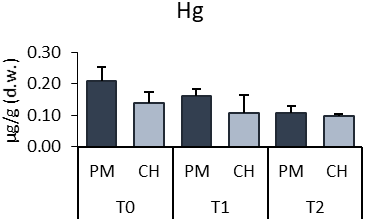


a

ab

b

*


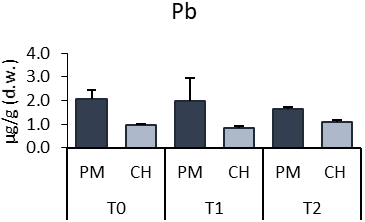


*

*


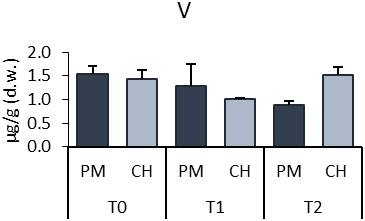


a

ab

b

AB

A

B


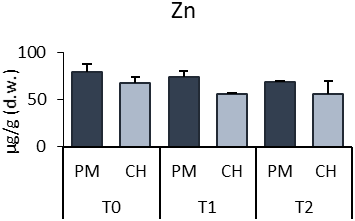


*

**1B**

**1C**

**1D)**

|  |  |  |  |  |  |  |  |  |  |  |  |  |  |
| --- | --- | --- | --- | --- | --- | --- | --- | --- | --- | --- | --- | --- | --- |
| VARIABLE |  | SITE | | |  | TIME | | |  | SITExTIME | | |  |
|  |  | dF | F | P |  | dF | F | P |  | dF | F | P |  |
| Al |  | 1 | 1.58 | N.S. |  | 2 | 0.93 | N.S. |  | 2 | 2.22 | N.S. |  |
| As |  | 1 | 0.03 | N.S. |  | 2 | 3.06 | N.S. |  | 2 | 0.51 | N.S. |  |
| Cd |  | 1 | 64.03 | p < 0.001 |  | 2 | 8.38 | p < 0.01 |  | 2 | 6.14 | p < 0.05 |  |
| Cr |  | 1 | 3.62 | N.S. |  | 2 | 1.27 | N.S. |  | 2 | 4.88 | N.S. |  |
| Cu |  | 1 | 5.59 | p < 0.05 |  | 2 | 2.97 | N.S. |  | 2 | 0.77 | N.S. |  |
| Fe |  | 1 | 5.44 | p < 0.05 |  | 2 | 1.35 | N.S. |  | 2 | 3.67 | N.S. |  |
| Hg |  | 1 | 7.21 | p < 0.05 |  | 2 | 6.08 | p < 0.05 |  | 2 | 1.15 | N.S. |  |
| Mn |  | 1 | 0.84 | N.S. |  | 2 | 1.98 | N.S. |  | 2 | 5.36 | N.S. |  |
| Ni |  | 1 | 1.17 | N.S. |  | 2 | 3.72 | N.S. |  | 2 | 2.64 | N.S. |  |
| Pb |  | 1 | 20.58 | p < 0.01 |  | 2 | 0.18 | N.S. |  | 2 | 0.96 | N.S. |  |
| V |  | 1 | 0.61 | N.S. |  | 2 | 3.79 | p < 0.05 |  | 2 | 6.55 | N.S. |  |
| Zn |  | 1 | 15.58 | p < 0.05 |  | 2 | 3.57 | N.S. |  | 2 | 0.37 | N.S. |  |
| PAHs LMW |  | 1 | 1.39 | N.S. |  | 2 | 0.25 | N.S. |  | 2 | 3.29 | N.S. |  |
| PAHs HMW |  | 1 | 2.82 | N.S. |  | 2 | 3.1 | N.S. |  | 2 | 0.03 | N.S. |  |
| PAHs tot |  | 1 | 0.11 | N.S. |  | 2 | 1.72 | N.S. |  | 2 | 2.12 | N.S. |  |
| HYDs tot |  | 1 | 1.49 | N.S. |  | 2 | 1.79 | N.S. |  | 2 | 0.094 | N.S. |  |
|  |  |  |  |  |  |  |  |  |  |  |  |  |  |
