# Supplementary material for: Long‐lasting effects of chronic exposure to chemical pollution on the hologenome of the Manila clam
Source: Evol Appl. 2021 Nov 27;14(12):2864–80. doi: 10.1111/eva.13319 (PMC8674894; doi:10.1111/eva.13319)
Supplement: Supplementary file 4 — File S4 [file EVA-14-2864-s004.docx]

**Biomarker responses**

Biomarker responses in *R. philippinarum* at each sampling time (T0, T1, T2) in the different sites (PM and CH). Data are given as mean values ± standard deviation, n = 5. Lower-case letters indicate significant differences of PM site among sampling times; upper-case letters indicate significant differences of CH site among sampling times; asterisks indicate significant differences between sites within the same sampling time.


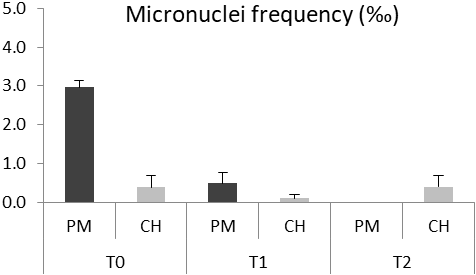


a

b

b

*


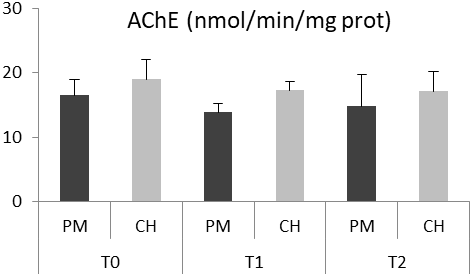


*


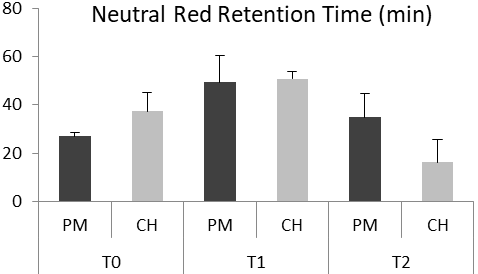


a

a

b

A

B

C


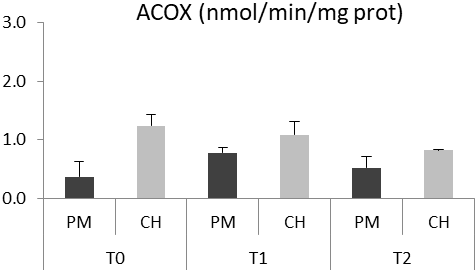


a

b

ab

A

AB

B

*

*

*


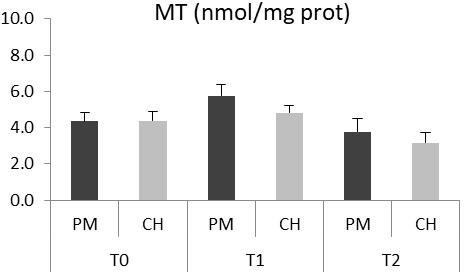


a

b

a

A

A

B

*


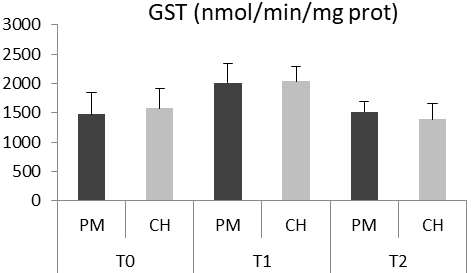


a

a

b

A

B

A


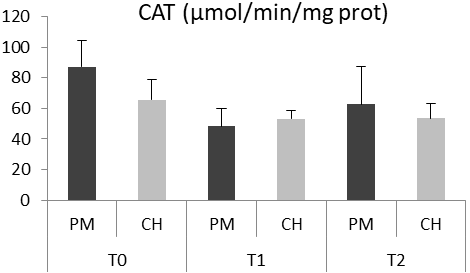


b

b

a


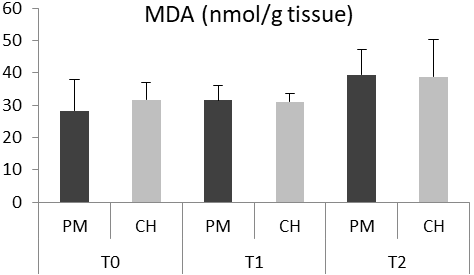


a

ab

b
