# Supplementary figures and images for: Long‐lasting effects of chronic exposure to chemical pollution on the hologenome of the Manila clam
Source: Evol Appl. 2021 Nov 27;14(12):2864–80. doi: 10.1111/eva.13319 (PMC8674894; doi:10.1111/eva.13319)

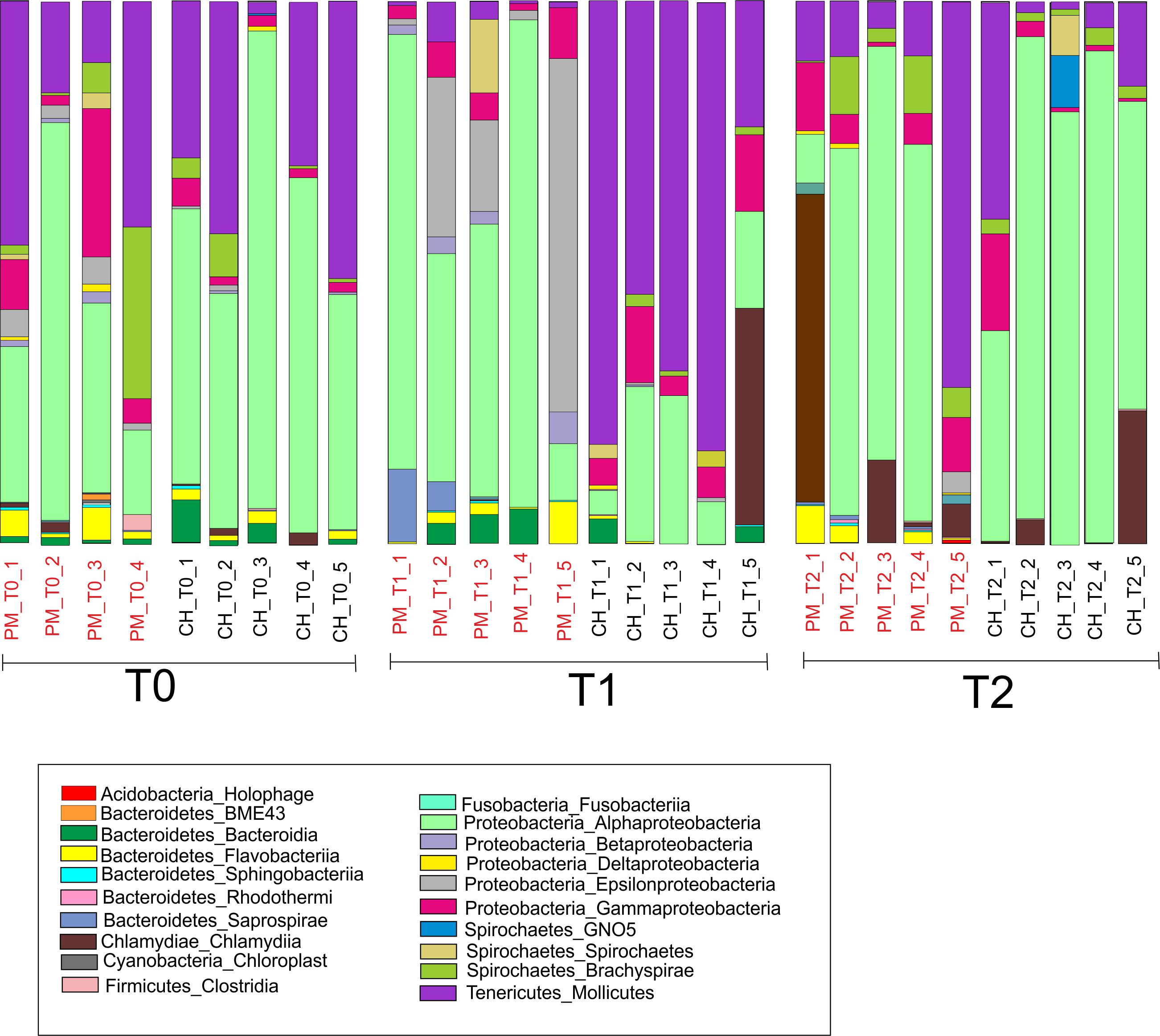

Supplement: Supplementary file 7 — File S7 [file EVA-14-2864-s008.jpg]

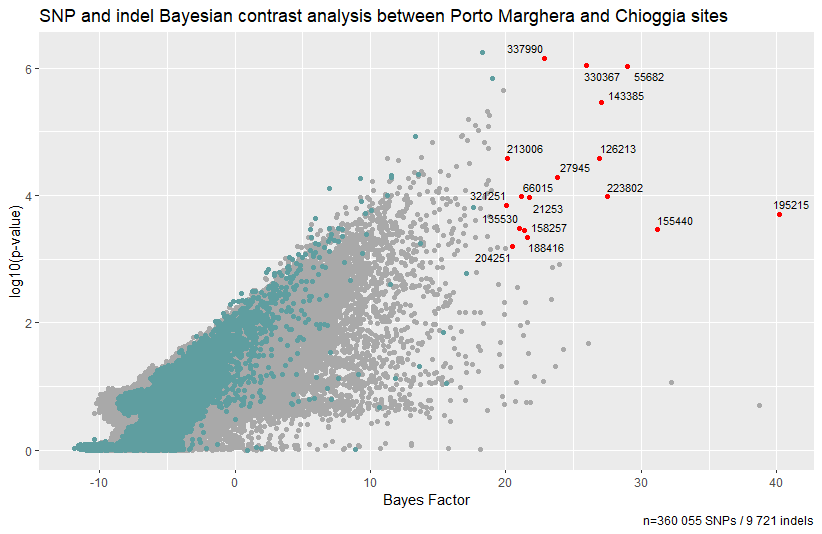

Supplement: Supplementary file 8 — File S8 [file EVA-14-2864-s005.png]
